# Pore-forming spider venom peptides show cytotoxicity to hyperpolarized cancer cells expressing K^+^ channels: A lentiviral vector approach

**DOI:** 10.1371/journal.pone.0215391

**Published:** 2019-04-12

**Authors:** Masayoshi Okada, Ernesto Ortiz, Gerardo Corzo, Lourival D. Possani

**Affiliations:** 1 Department of Medical Life Science, College of Life Science, Kurashiki University of Science and the Arts, Kurashiki, Okayama, Japan; 2 Departamento de Medicina Molecular y Bioprocesos, Instituto de Biotecnología, Universidad Nacional Autónoma de México, Cuernavaca, Morelos, Mexico; Weizmann Institute of Science, ISRAEL

## Abstract

Recent studies demonstrated the upregulation of K^+^ channels in cancer cells. We have previously found that a pore-forming peptide LaFr26, purified from the venom of the *Lachesana sp* spider, was selectively incorporated into K^+^ channel expressing hyperpolarized cells. Therefore, it is expected that this peptide would have selective cytotoxicity to hyperpolarized cancer cells. Here we have tested whether LaFr26 and its related peptide, oxyopinin-2b, are selectively cytotoxic to K^+^ channel expressing cancer cells. These peptides were cytotoxic to the cells, of which resting membrane potential was hyperpolarized. The vulnerabilities of K^+^ channel-expressing cell lines correlated with their resting membrane potential. They were cytotoxic to lung cancer cell lines LX22 and BEN, which endogenously expressed K^+^ current. Contrastingly, these peptides were ineffective to glioblastoma cell lines, U87 and T98G, of which membrane potentials were depolarized. Peptides have a drawback, i.e. poor drug-delivery, that hinders their potential use as medicine. To overcome this drawback, we prepared lentiviral vectors that can express these pore-forming peptides and tested the cytotoxicity to K^+^ channel expressing cells. The transduction with these lentiviral vectors showed autotoxic activity to the channel expressing cells. Our study provides the basis for a new oncolytic viral therapy.

## Introduction

Recent studies have shown that some K^+^ channels are upregulated in cancer cells [1, 2]. For instance, pathological examinations showed upregulation of the two-pore domain type K^+^ channel, TREK-1 [3], in prostate cancer and of the inwardly rectifying K^+^ channel, Kir2.1, in lung cancer [4], human ether-a-go-go, HERG, in neuroblastoma [5, 6] whereas surrounding normal cells did not express them. The expression levels of Kir4.1 channel in glioma cells were correlated with clinical stage and chemoresistance [7]. The expression of HERG channel was implicated in cell proliferation and transformation [5]. The upregulated K^+^ current seemed to play a role in cell proliferation, migration, and cell cycle progression [1, 2].

Arachnid venoms contain pore-forming peptides that are incorporated into the cell membrane where they assemble to form pores. The formed pores conduct ions like ionophores, resulting in several biological activities, e.g., anti-microbial [8], hemolytic [9], and pain-inducing effects [10]. Previously, we have purified a 69 amino acid peptide, LaFr26, from the venom of a spider, *Lachesana sp* [11]. An identical peptide was also purified from another species, *Lachesana tarabaevi*, and named cyto-insectotoxin 1a (CIT1a) [12]. LaFr26 (CIT1a) is one of the peptides encoded by a gene family, which consists of similar pore-forming venom peptides purified from various spiders [11]. Among them, oxyopinin-1 and -2b, which were purified from wolf spider, had similar hemolytic activity [13]. Interestingly, LaFr26 has a unique feature of selective incorporation into 293T cells that expressed the inwardly rectifying K^+^ channel, Kir2.1, relative to the control 293T cells [11]. This selectivity was due to the basic amino acids of the peptide and hyperpolarized membrane potential of K^+^ channel-expressing cells. Therefore, it is possible that the pore-forming peptide might be selectively incorporated into cancer cells, thereby being cytotoxic only to cancer cells.

In spite of these promising features, the pore-forming venom peptides have a drawback, i.e. poor drug-delivery. If they are orally administrated, they will be digested in the gastrointestinal tract. If they are intravenously injected, they would be quickly diluted and incorporated into other cells. Intratumoral injection seems to be the only way, but frequent intratumoral injection is difficult and unsafe. One option to overcome this drawback is the use of lentiviral vector, which is based on the human immunodeficiency virus and has high infectious and long-lasting expression abilities [14]. If the lentiviral vector transduces the genes coding for the venom peptides, it is expected that the transduced tumor cells would express the peptides and kill themselves.

Here we tested these possibilities: selective toxicity to K^+^ channel expressing cells and viral vector-mediated toxicity. We found hyperpolarized cell-selective cytotoxicity of chemically synthesized LaFr26 and oxyopinin-2b and cytotoxicity of the lentiviral vectors expressing these peptides.

## Materials and methods

### Materials and cell culture

LaFr26 (CIT1a), GFFGNTWKKIKGKADKIMLKKAVKIMVKKEGISKEEAQAKVDAMSKKQIRLYLLKYYGKKALQKASEKL, was chemically synthesized (Peptide2.0, VA, USA), as shown in our previous study [11]. The purity was analyzed with a C_18_ column and was 96.1%. Oxyopinin-2b (GKFSGFAKILKSIAKFFKGVGKVRKGFKEASDLDKNQ) was synthesized by a solid-phase method using the Fmoc methodology on an Applied Biosystems 433 A peptide synthesizer. Fmoc-Gln(tBu)-PEG resin (Watanabe Ltd., Hiroshima, Japan) was used to provide a free carboxyl at the C-terminus. After synthesis, cleavage and deprotection of peptide from resin were performed according to a previous report [13]. The synthetic peptide was dissolved in a 30% aqueous acetonitrile solution and separated by reverse-phase HPLC on a semipreparative C_18_ column (10 x 250 mm, Nacalai Tesque, Japan). The mass identity of synthetic peptides was verified by mass spectrometry.

Preparation of stable cell lines that express Kir2.1, TREK-1, HERG-1, and GFP were described in a previous report [15]. BEN and LX22 cells were kindly gifted by Dr. John Laterra [16], and glioblastoma cells were purchased from (Japanese Collection of Research Bioresources Cell Bank, Osaka, Japan). Cells were maintained in Dulbecco’s modified Eagle’s medium containing 10% fetal bovine serum and 1% penicillin/streptomycin. Cytotoxicity was measured with cells grown in a 96-well plate using the Cell Counting Kit-8 (Dojindo, Tokyo, Japan) according to the manufacturer’s instructions.

### Construction of the gene cassettes for spider peptides’ expression and preparation of the lentiviral vectors

Gene constructions were devised for the expression of the recombinant spider toxins in tumor cells upon transduction of the lentiviral vectors. The expression cassettes were designed to contain transcriptional fusions between the reporter green fluorescent protein (hrGFP) gene and the spider peptide genes, linked by an internal ribosome entry site (IRES2). The hrGFP II-IRES2 fragment was amplified by PCR using vector CS-ß-actinP-hrGFP-IRES-Kir2.1 as a template [17]. The cDNAs of the LaFr26 and Oxyopinin-2b were generated by reverse translation from the mature peptides, and the codons were optimized and harmonized for optimal expression in mammalian cells. The peptide genes were preceded by a Kozak site and the sequence coding for the *Gaussia* luciferase signal peptide (GLucSP) for proper secretion [18], ended with two stop codons (TAA-TAG) and were flanked by the MscI and BamHI cloning sites. The MscI-Kozak-GLucSP-LaFr26-BamHI and MscI-Kozak-GLucSP-Oxyopinin-2b-BamHI genes were assembled by recursive PCR from synthetic oligonucleotides, cloned into the pBluescript KS(+) vector and verified by sequencing. The cloned genes for the spider peptides were re-amplified by PCR to generate an overlapping region with the previously amplified hrGFP II-IRES2 fragment. The full constructions were then assembled by recursive PCR taking advantage of this overlapping region. The products with the expected sizes were cloned in pBluescript KS (+) vector and verified by sequencing. The correct cassettes, BamHI-hrGFP II-IRES2-GLucSP-LaFr26-BamHI, and BamHI-hrGFP II-IRES2-GLucSP-Oxyopinin-2b-BamHI were obtained by BamHI digestion, purified and cloned into the lentiviral shuttle vector CS-βactinP, which was modified from CS-CDF-CG-RRE (donated by Dr. Miyoshi, Riken, Ibaraki, Japan). Envelop protein was pseudotyped with VSV-g protein and lentiviral vectors were prepared as described previously [17]. Three vectors were used as control: Lv-GFP, Lv-mCherry, and Lv-ROMK express GFP, mCherry, and ROMK(Kir1.1) and GFP, respectively.

To detect the secreted peptide in the media, we collected the media of the cells transduced with Lv-LaFr26 and control vector, Lv-ROMK, 48 h after transduction. Then the media (100 μL) were centrifuged at 1,500 rpm for 3 min and the supernatant was again centrifuged at 14,000 rpm for 1 min with a microfuge. The supernatant was analyzed with a HiTrap SP HP cation exchange column (GE Healthcare, Pittsburgh, PA). Peptides were eluted with a gradient of NaCl from 200 to 2,000 mM in 10 mM Tris-HCl (pH 7.4), monitoring A_230 nm_ with a UV detector (JASCO, Tokyo, Japan).

### Patch-clamp recordings

Cells grown on a small cover glass (3 × 18 mm) were placed in a recording chamber. Whole-cell currents were recorded in Tyrode solution using an Axopatch 200B amplifier (Axon Instruments, Foster City, CA) at 25°C [15]. Tyrode solution contained (in mM): NaCl 140, KCl 5.4, NaH_2_PO_4_ 0.33, CaCl_2_ 2, MgCl_2_ 1, HEPES 5, and glucose 5.5 (pH 7.4 adjusted with NaOH). Patch pipettes pulled from borosilicate glass (Narishige, Tokyo, Japan) were filled with an internal solution containing (in mM): K-aspartate 66, KCl 71.5, KH_2_PO_4_ 1, EGTA 5, HEPES 5, and MgATP 3 (pH 7.4 adjusted with KOH). Recordings were digitized at 10 kHz, and low-pass filtered at 2 kHz. TREK-like current was evoked by step pulses as shown in the Figure. Resting membrane potential was measured in a whole-cell current-clamp configuration. The whole-cell membrane and access resistance were measured with a depolarizing step pulse from the holding potential (-70 mV) to -50 mV.

### Statistical analysis

Data are given as the mean ± SEM. The data obtained from two groups were analyzed statistically with Student's t-test, and those from various groups were analyzed by one-way analysis of variance (ANOVA) followed by Student’s *t*-test. A *p* value of < 0.05 was considered significant. *, **, and *** indicate *p* < 0.05, 0.01, and 0.005, respectively.

## Results

### Hyperpolarization-dependent cytotoxicity to the model cells that express K^+^ channels

**Fig 1:**
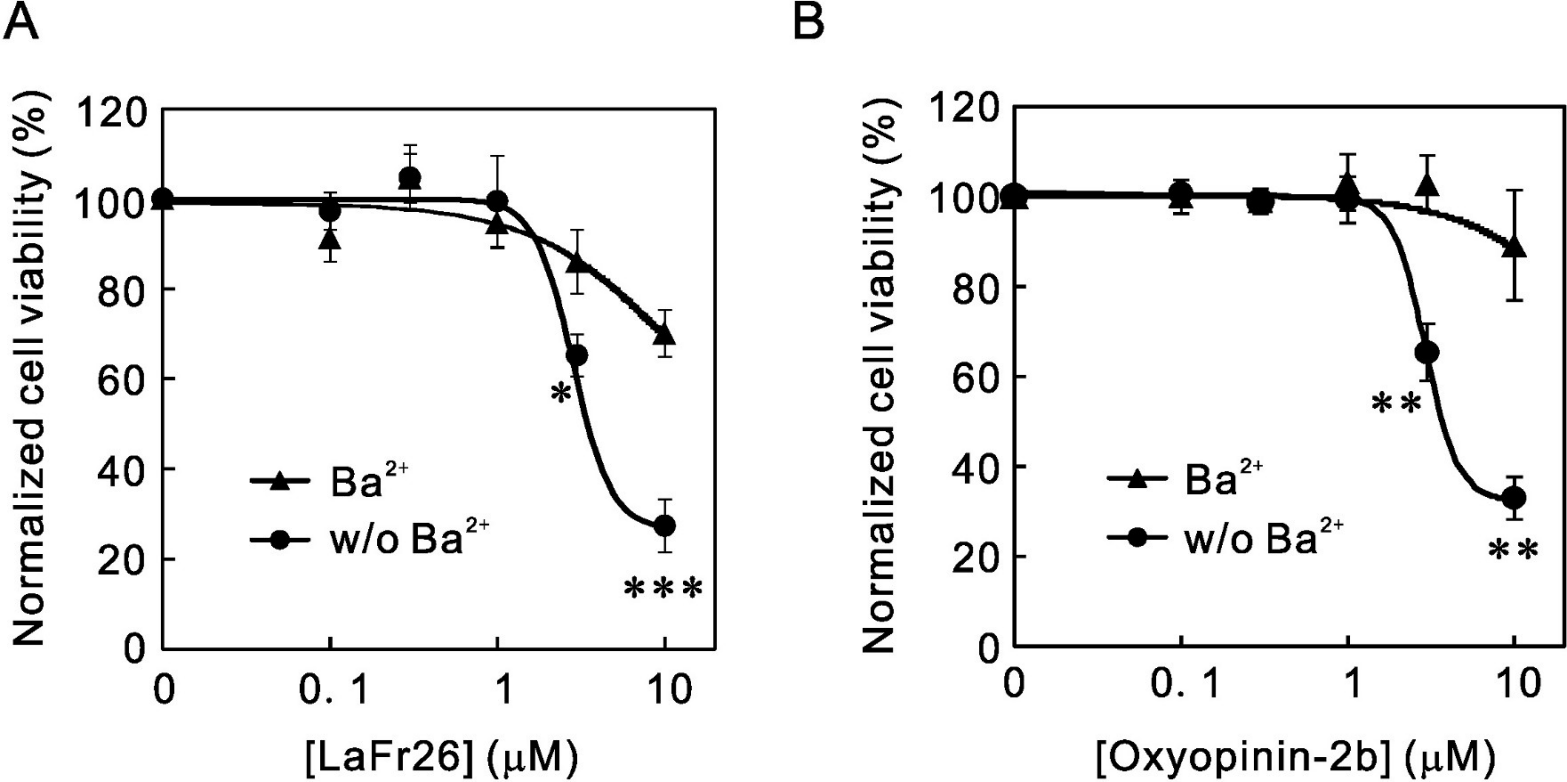
K^+^ current-dependent cytotoxicity of LaFr26 and oxyopinin-2b. (A and B) LaFr26 (A) and oxyopinin-2b (B) were added to the media of 293T cells that stably expressed Kir2.1 at indicated concentrations in the presence (Ba^2+^) or absence (w/o Ba^2+^) of 0.3 mM BaCl_2_. Cell viabilities were measured 30 min after addition (Student’s *t*-test, Ba^2+^ vs w/o Ba^2+^, n = 4).

We first examined cytotoxicity of chemically synthesized LaFr26 and oxyopinin-2b on 293T cells stably expressing Kir2.1 (56–3). 293T cells were derived from human embryonic kidney, not from the tumor, and the 56–3 cell line was prepared by transfection with lentiviral vectors containing the gene for the Kir2.1 channels’ expression [15]. We added the peptides to the cell culture media and incubated for 30 min. Cell viability was colorimetrically determined with the Cell Counting Kit-8, which measures the dehydrogenase activities of living cells. The addition of LaFr26 decreased the cell viability of Kir2.1 expressing cells, 56–3, in a concentration-dependent way (Fig 1A). Similarly, oxyopinin-2b also exhibited cytotoxicity in a concentration-dependent way (Fig 1B). To test whether this cytotoxicity is dependent on K^+^ current, we added a selective blocker for Kir2.1, Ba^2+^, to the medium immediately before the addition of the peptides. The addition of the blocker significantly inhibited cytotoxicity (Fig 1A and 1B), indicating the K^+^ channel current-dependency of the cytotoxicity. The relationship between cytotoxicity and LaFr26 and oxyopinin-2b concentrations were fitted by Hill’s equation and the EC_50_ was estimated to be 2.96 and 3.01 μM, respectively.

**Fig 2:**
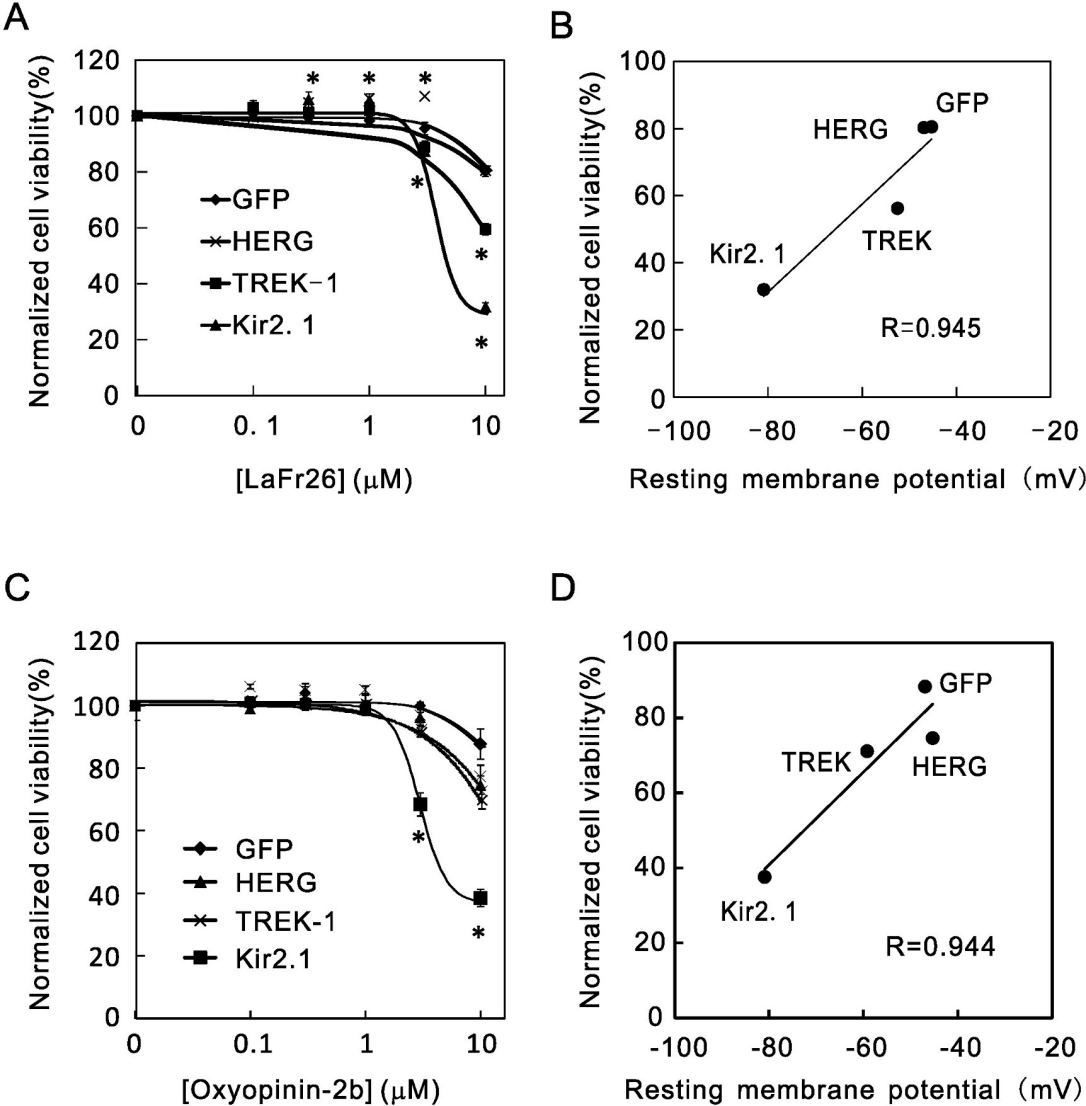
Resting membrane potential-dependency of cytotoxicity of the venom peptides. (A) LaFr26 was added to the media of 293T cells that stably express GFP, HERG-1, TREK-1, or Kir2.1, and cell viabilities were measured (ANOVA followed Student’s *t*-test, vs 0 μM, n = 6). (B) A significant correlation was found between cell viabilities at 10 μM and the resting membrane potential measured with whole-cell patch-clamp recordings. (C) Oxyopinin-2b was added to the media of these cells (n = 6). (D) Cell viability at 10 μM again correlated with the resting membrane potentials.

K^+^ efflux through the channel resulted in hyperpolarization of membrane potential, and therefore the cytotoxicity might be directly dependent on membrane potential. To test this, we examined the cytotoxicity to three cell lines that express different K^+^ channels, i.e., Kir2.1, TWIK-related K^+^ channel (TREK-1), and human ether-a-go-related gene (HERG) K^+^ channel. Each K^+^ channel opens and conducts K^+^ current according to their own voltage-dependency; their open probabilities at resting status are also different. For this reason, the resting membrane potentials of these cell lines were different: Kir2.1 (-80.9 mV) > TREK-1 (-59.2 mV) > HERG (-45.3 mV) = Control (expressing GFP, -46. 9 mV). If LaFr26 and oxyopinin-2b are selectively incorporated into cells with hyperpolarized resting membrane potential, the cytotoxicity should be different depending on the resting membrane potentials. As expected, the vulnerabilities to the LaFr26 were different (Fig 2A). The cells expressing Kir2.1, which resting membrane potential was the most hyperpolarized, were the most vulnerable; the cells expressing HERG-1, which membrane potential was similar to that of the GFP control, were the least vulnerable. The resting membrane potential and the vulnerability of the TREK-1 expressing cells were in the middle. The vulnerabilities correlated with the resting membrane potential; the correlation coefficient R = 0.945 (Fig 2B). A similar relationship was obtained with oxyopinin-2b (R = 0.944, Fig 2C and 2D).

### Cytotoxicity to cancer cells

**Fig 3:**
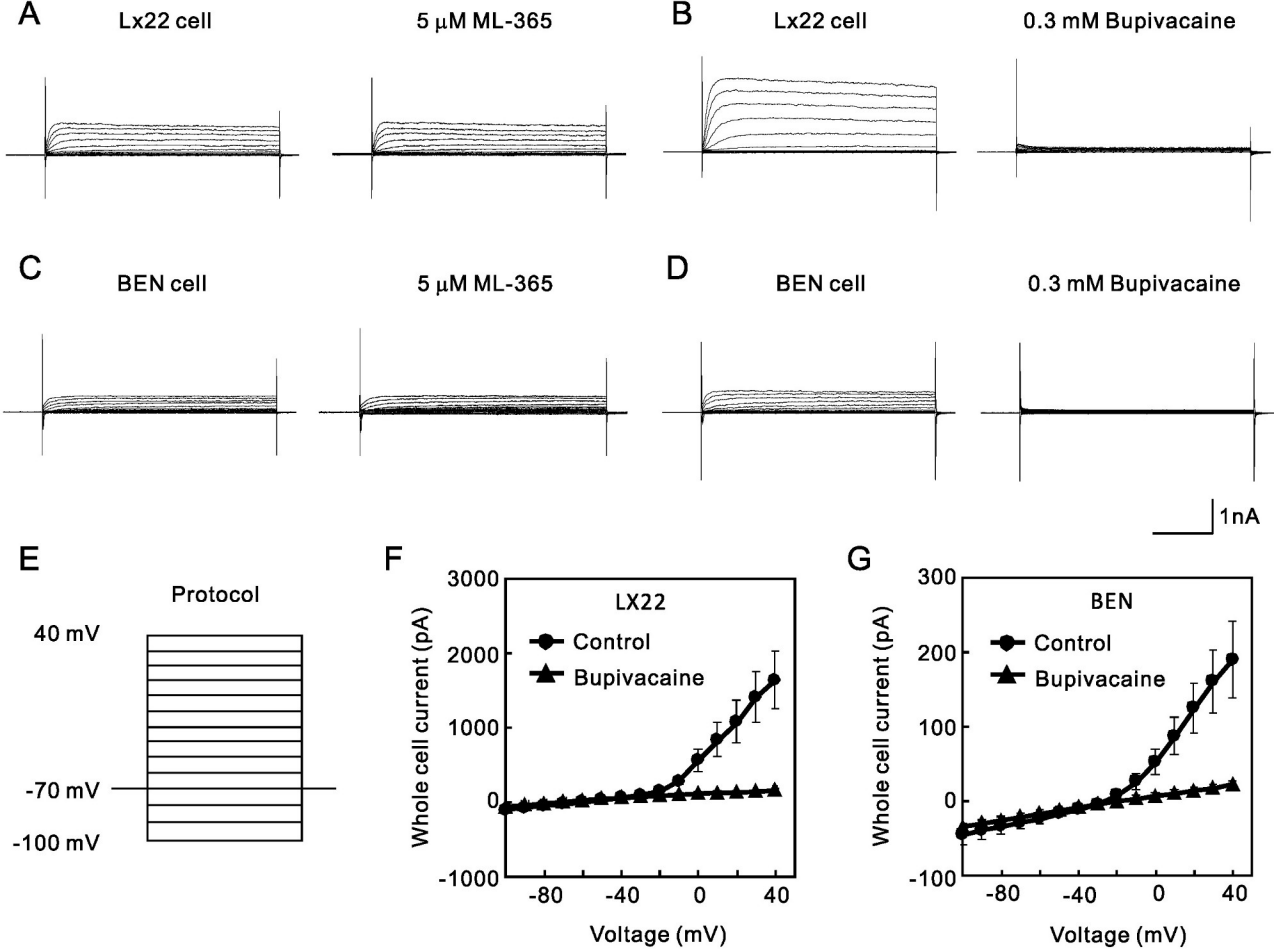
Endogenous expression of TREK-like current in the lung cancer LX22 and BEN cells. After whole-cell access was made from LX22 and BEN cells, outward rectified currents were evoked by the step pulses indicated as protocol (E). (A and B) LX22 cells expressed currents resistant to 5 μM ML365 and sensitive to bupivacaine, which were similar to TREK-1 current. (C and D) BEN cells also expressed 0.3 mM bupivacaine-sensitive current. (F and G) I-V relationship of the whole cell current expressed in LX22 and BEN cells. The I-V relationship indicates the outward rectification of the current and sensitivity to bupivacaine.

A recent study showed increased expression of K^+^ channel in malignant small lung cancer cell line, LX22, and lung carcinoma cell line, BEN [16], therefore we next examined the cytotoxicity of the pore-forming peptides to these cell lines. We first confirmed the endogenous expression of K^+^ channel current in these lung cancer cell lines with whole-cell patch clamp recordings. To evoke the TREK-like current, cells were initially voltage-clamped at -70 mV, and step pulses from -100 to 40 mV (0.4 sec) were applied. As expected, LX22 cells expressed TREK-like outward-rectified current (Fig 3). These cells were reported to express two-pore domain K^+^ channels [16], and therefore we tested the effect of blockers, ML365 and bupivacaine. Whereas ML365 had little effect on the currents (Fig 3A), 0.3 mM bupivacaine blocked the current almost completely (Fig 3B and 3F). Similarly, BEN cells also expressed ML365-resistant (Fig 3C) and bupivacaine-sensitiveTREK-like current (Fig 3D and 3G).

**Fig 4:**
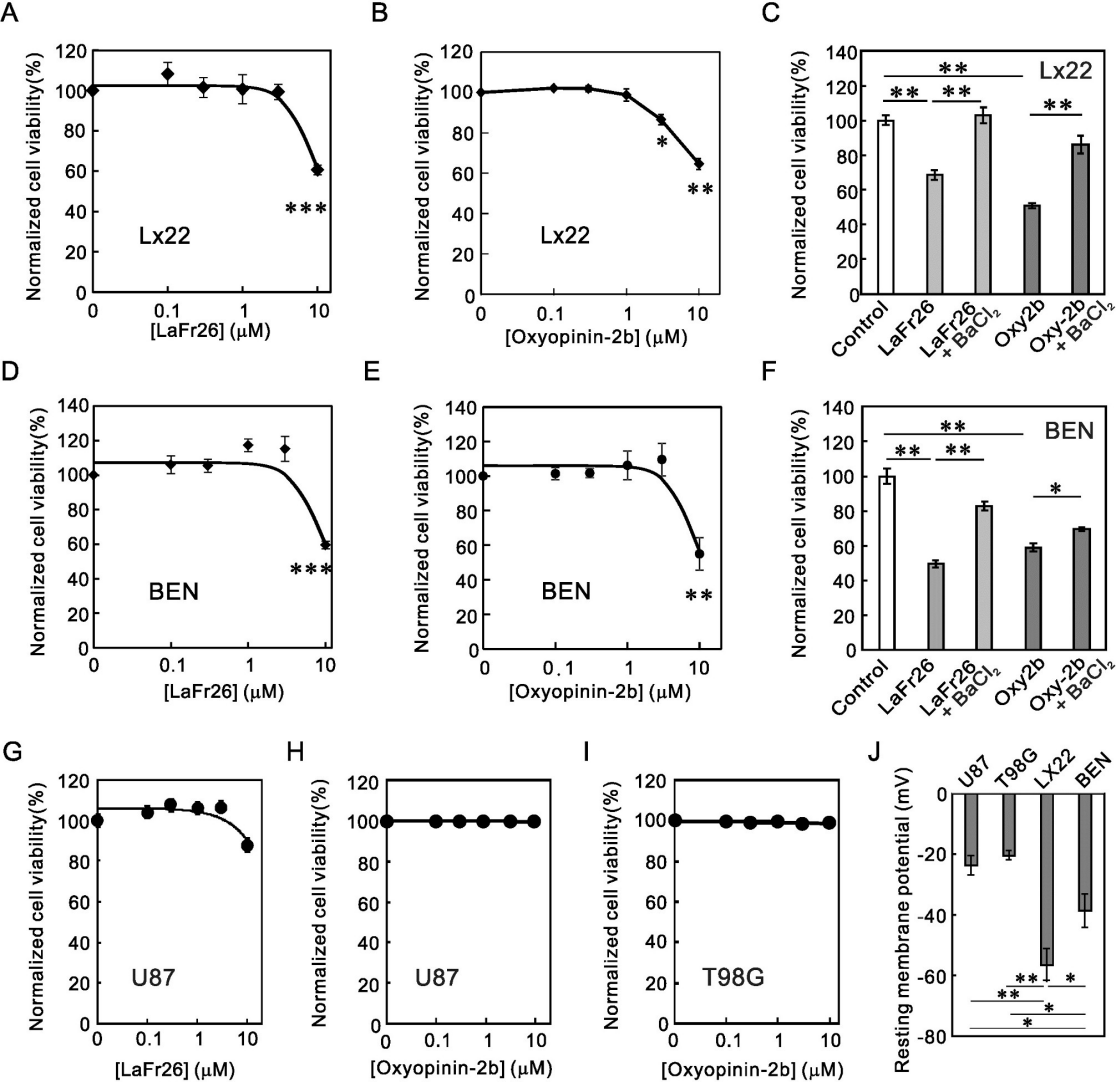
Cytotoxicity of LaFr26 and oxyopinin-2b to LX22 and BEN cells, but not to U87 or T98G cells. (A and B) LaFr26 and oxyopinin-2b were added to the media of LX22 cells, and the cell viabilities were measured 30 min after (ANOVA followed Student’s *t*-test, vs 0 μM, n = 6). (C) LX22 cells were incubated with these peptides (10 μM) in the presence or absence of a K^+^ channel blocker, BaCl_2_ (1 mM) and viability were measured. The addition of Ba^2+^ inhibited the cytotoxicity of peptides, indicating the K^+^ channel current dependency. (D and E) LaFr26 and oxyopinin-2b were added to the media of BEN cells (n = 6). (F) The addition of Ba^2+^ again inhibited the cytotoxicity to BEN cells. (G, H, and I) LaFr26 and oxyopinin-2b were added to the media of U87 or T98G cells. No cytotoxicity was observed. (J) Resting membrane potentials of U87, T98G, LX22, and BEN cells (n = 10, 5, 7, and 5).

To test the cytotoxicity of LaFr26 and oxyopinin-2b to these cancer cells, these peptides were added to the medium of the cells cultivated in a 96-well plate. As expected, these peptides showed cytotoxicity to LX22 (Fig 4A and 4B) and BEN (Fig 4D and 4E) cells. We tried to confirm the K^+^ channel current dependency by blocking the current with bupivacaine. The addition of bupivacaine, however, decreased cell viability, probably because bupivacaine has its own independent cytotoxic effect [19, 20]. Therefore, to block the TREK-like current, we added 1 mM BaCl_2_, which was shown to block TREK-1 channel current, and incubated LX22 and BEN cells with 10 μM LaFr26 or oxyopinin-2b. As expected, the addition of BaCl_2_ significantly inhibited the cytotoxicity of LaFr26 and oxyopinin-2b (Fig 4C and 4F).

To examine whether these pore-forming peptides are cytotoxic or not to another type of cancer cells, we incubated glioblastoma cell lines, U87 and T98G, with LaFr26 or oxyopinin-2b and measured viability with CCK-8 (Fig 4G, 4H and 4I). Unexpectedly, these cells were resistant to the peptides even at 10 μM. To reveal the reason for this difference in vulnerability, we measured the resting membrane potentials of these cells by whole-cell patch-clamp recording. The membrane potentials were depolarized in these peptide-resistant glioblastoma cells (Fig 4J). In contrast, those of LX22 and BEN cells were hyperpolarized. This again suggests the resting membrane potential-dependency of the cytotoxicity elicited by the pore-forming peptides. In addition, this difference suggests their usefulness and limitation: effective only to hyperpolarized cancer cells.

### Cytotoxicity of lentiviral vectors expressing LaFr26 and oxyopinin-2b on K^+^ channel expressing cells

**Fig 5:**
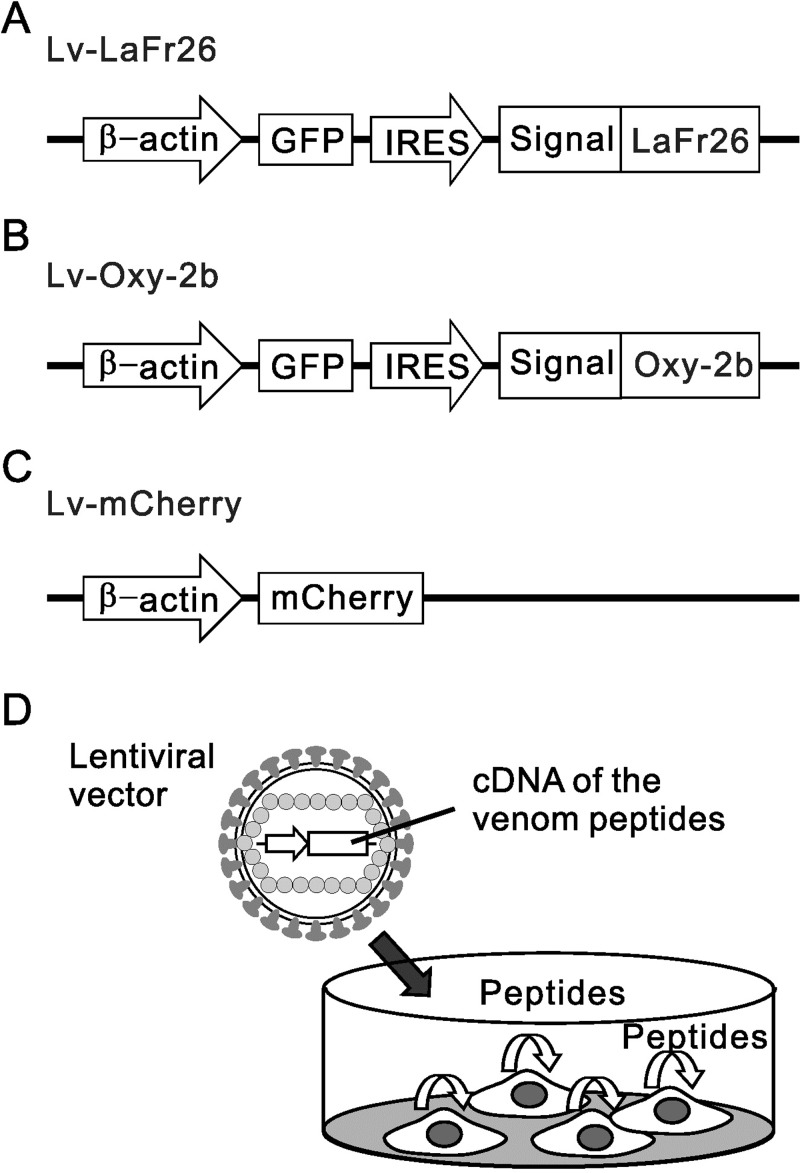
Schematic illustration of the designed lentiviral vectors used to transduce the cells with the genetic constructions for the expression of LaFr26, oxyopinin-2b, and mCherry. Lv-LaFr26 (A) and Lv-Oxy-2b (B) carry IRES for the bicistronic coexpression of GFP and the spider peptides, which were designed to be secreted byLaFr26 or oxyopinin-2b. (C) Structure of Lv-mCherry. The expression cassettes were inserted in the downstream of RNA packaging signal between two long terminal repeats. (β-actin, promoter of chick β-actin used for the coexpression; GFP, green fluorescent protein gene; IRES, internal ribosomal entry site for the bicistronic coexpression; Signal, *Gaussia* luciferase signal peptide; mCherry, mCherry gene) (D) Schematic illustration of the autotoxic expression of the venom peptides with Lv-LaFr26 and Lv-Oxy-2b.

Despite the therapeutic potential, peptide agents still have an unresolved drawback concerning their delivery to the tumor. To overcome this drawback, we prepared lentiviral vectors that can transduce the LaFr26 or oxyopinin-2b genes for their heterologous expression in the infected tumor cells (Fig 5). A strategy using an internal ribosome entry site (IRES) for the bicistronic coexpression of the green fluorescent protein (GFP) was implemented, which enabled the detection of the transduced cells with fluorescence. The genes coding for LaFr26 and oxyopinin-2b were successfully assembled by recursive PCR. Their sequences were optimized for their expression in mammalian cells. Since LaFr26 and oxyopinin-2b are secreted venom peptides, they should have their own spider signal peptides in their N-termini. But they are purified as mature peptides from the venom, so we do not have any information regarding their native signal peptides. Moreover, the cytotoxic effect was observed when the synthetic LaFr26 and oxyopinin-2b were applied extracellularly. Therefore, a system that can direct its secretion was mandatory. The spider venom peptides were expressed as a fusion peptide with the *Gaussia princeps* luciferase signal peptide (Fig 5), a short 17 amino acid sequence that directs the expressed peptides to secretion and that has been shown to work adequately in mammalian cells [18]. The signal peptides are normally cleaved upon entry of the nascent peptide to the endoplasmic reticulum during translation, so they do not interfere with the function of the secreted proteins. The whole constructions were placed under the control of the strong chicken β-actin promoter, with which we have successfully expressed various K^+^ channels before [15]. The lentiviral vectors containing the LaFr26 or oxyopinin-2b following the GFP-IRES were designated as Lv-LaFr26 and Lv-Oxy-2b, respectively. The viral vector that expressed the red fluorescent protein mCherry only was designated as Lv-mCherry (Fig 5C), which served as a control. We also prepared Lv-GFP, which expresses GFP, and Lv-ROMK, which co-expresses Kir1.1(ROMK, KCNJ1) and GFP as control.

**Fig 6:**
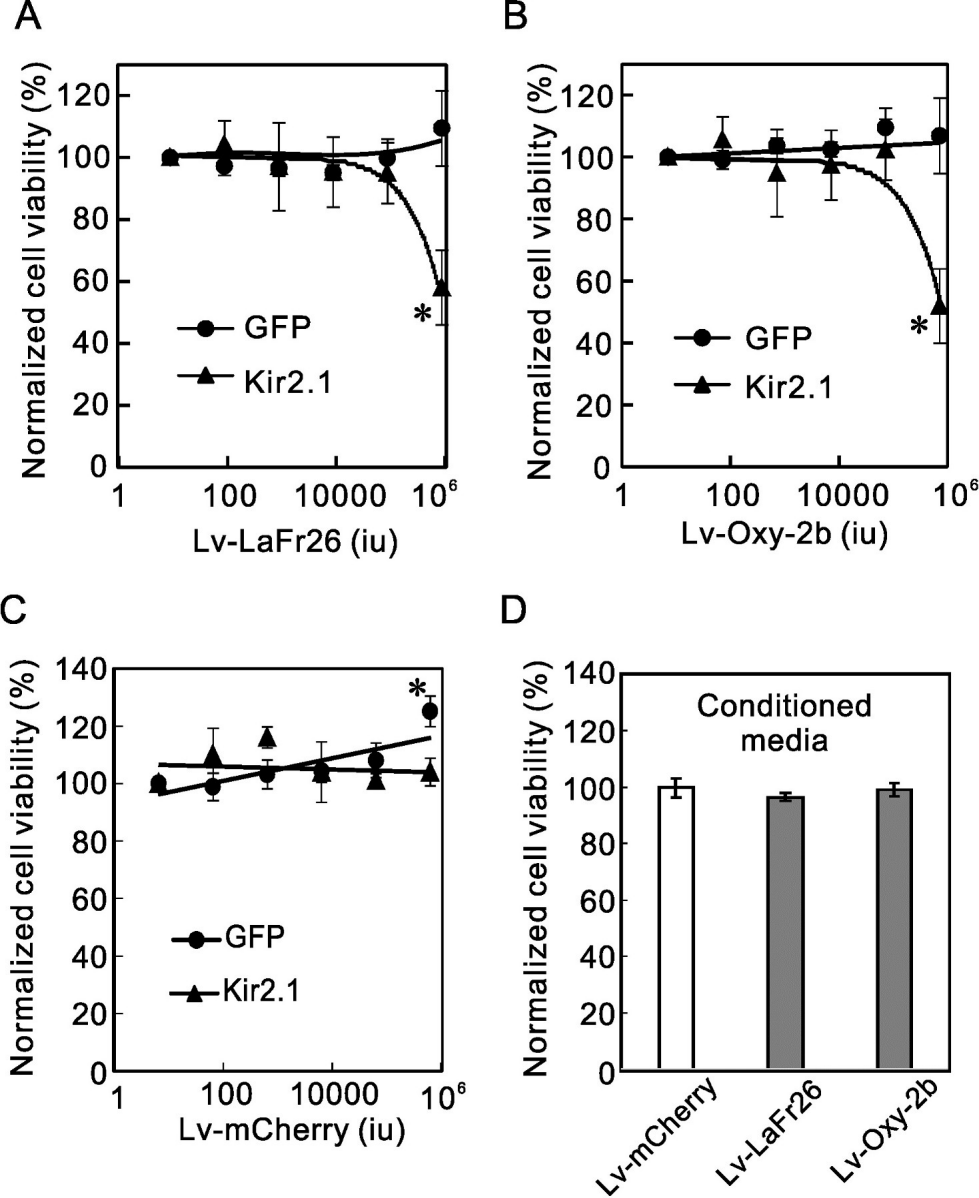
K^+^ channel current-dependent cytotoxicity Lv-LaFr26- and Lv-Oxy-2b-transduced cells. (A, B, and C) Lv-LaFr26, Lv-Oxy-2b, and Lv-mCherry were added to the media of 293T cells that stably express GFP or Kir2.1 and the cell viabilities were measured 72 h after addition. Both Lv-LaFr26 and Lv-Oxy-2b transductions resulted in cytotoxicity only to Kir2.1-expressing cells (A and B). (C) Contrastingly, Lv-mCherry infection did not lead to any cytotoxicity to GFP- or Kir2.1-expressing cells (n = 4). (D) 56–3 cells were incubated with conditioned media from the cells transduced with Lv-mCherry, Lv-LaFr26, and Lv-Oxy-2b. Cell viabilities were measured after 24 h (n = 8, 4, and 4).

We added various amounts of lentiviral vectors to the media of the Kir2.1 expressing 56–3 cells and incubated them for 24, 48, and 72 h after the addition. We subsequently measured the cell viability with a cell counting kit and found no significant decrease in viability up to 48 h after addition. Lv-LaFr26 (Fig 6A) and Lv-Oxy-2b (Fig 6B) treated Kir2.1-expressing cells showed a significant decrease in viability at 72 h after addition. Contrastingly, Lv-LaFr26 and Lv-Oxy-2b had no effect on GFP expressing cells even after 72 h, confirming the K^+^ channel current (i.e., hyperpolarization)-dependency of cytotoxicity. This K^+^ current-dependent cytotoxicity is not due to the general toxicity of the lentiviral vector infection because Lv-mCherry had no cytotoxicity even to Kir2.1 expressing cells (Fig 6C).

**Fig 7:**
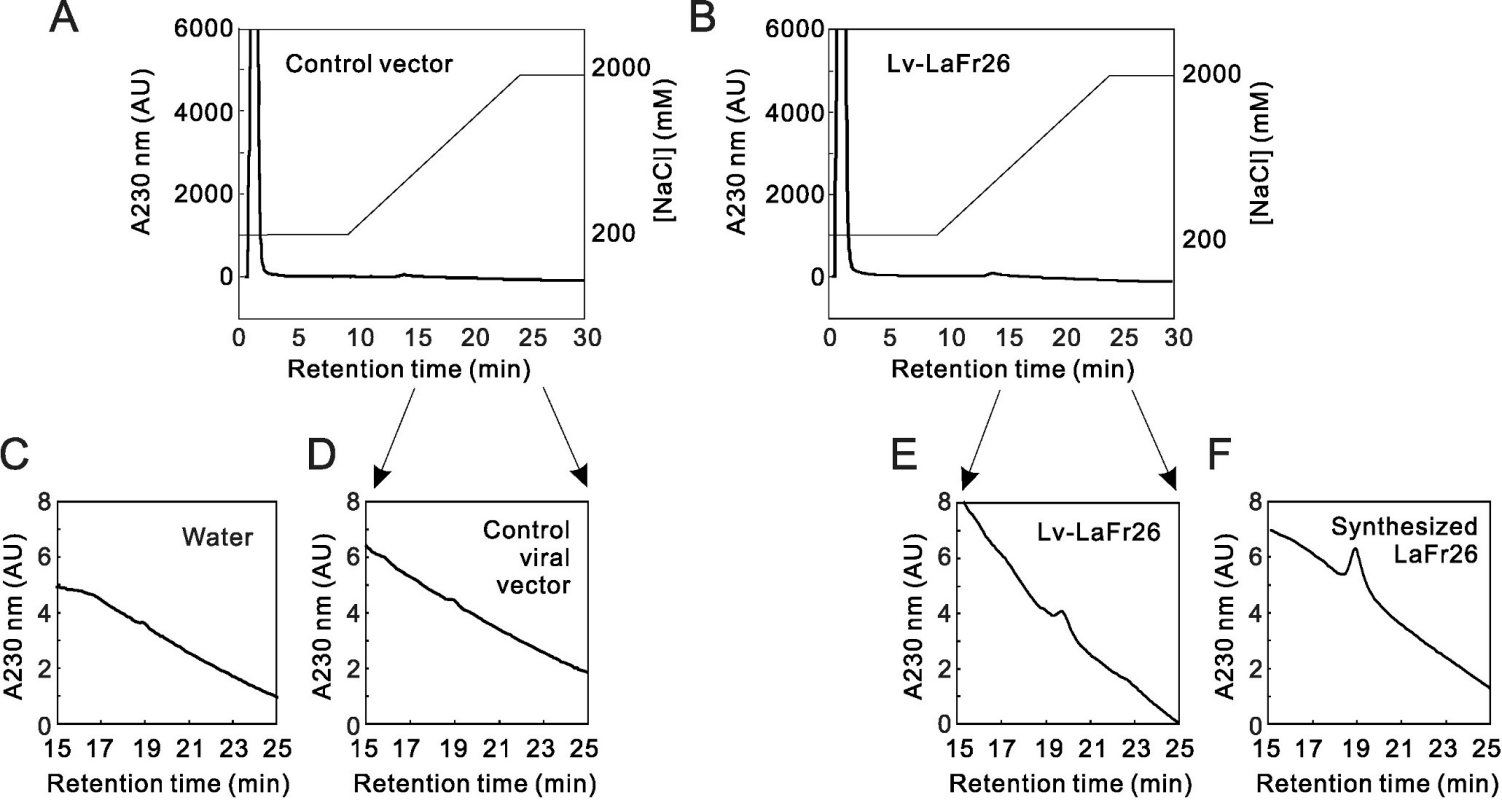
Chromatogram of media of control vector and Lv-LaFr26 transduced cells. (A and B) The supernatant (100 μL) of media collected 48 h after viral transduction were fractionated by a cation-exchange column. Ordinate indicates the A_230_ nm (arbitral unit of HPLC UV-detector) and NaCl concentration (mM). (C, D, E, F) Chromatogram from 15 to 25 min was enlarged.

To detect the secreted peptide in the media, we collected the media from cells transduced with Lv-LaFr26- and control Lv-ROMK-transduced 293T cells 48 h after transduction. The collected media were analyzed with a HiTrap SP HP cation exchange column. We first injected the chemically synthesized LaFr26 (1 μM), monitoring A_230 nm_ (Fig 7). The peak height was too small in the upper panels, so the chromatogram is shown enlarged in lower panels. We found a peak with a retention time of 18.9 min on the declining baseline (Fig 7F). Next, the injection of the medium from Lv-LaFr26-transduced cells resulted in a peak at 19.8 min (Fig 7E), showing a similar, though a bit different in the retention time (see discussion). Injection of the medium from control viral vector-transduced cells resulted in a faint peak at around 19 min (Fig 7D), which was also detected with an injection of distilled water (Fig 7C). These results suggest the successful secretion of LaFr26 to the supernatant.

But, unexpectedly, the peak height in the chromatography of the medium sample was smaller than that of 1 μM chemically synthesized LaFr26 (compare Fig 7E and 7F). Since LaFr26 showed substantially no cytotoxicity at sub-μM range (Fig 1A), we assume the concentration in the medium could be too low to exhibit cytotoxicity. To test this lack of cytotoxicity, we collected the conditioned media of the 48-h cultivated 293T cells transduced by Lv-LaFr26 and Lv-oxy-2b. Subsequently, the conditioned media were added to 96-well plates, in which 56–3 cells were grown, and their viability was measured viability after 24 h. There was no difference in the viability compared with that of control (Fig 6D). Therefore, it is likely that the peptides were incorporated into surrounding cells immediately after secretion and thereby were not recovered from the supernatant (see discussion).

**Fig 8:**
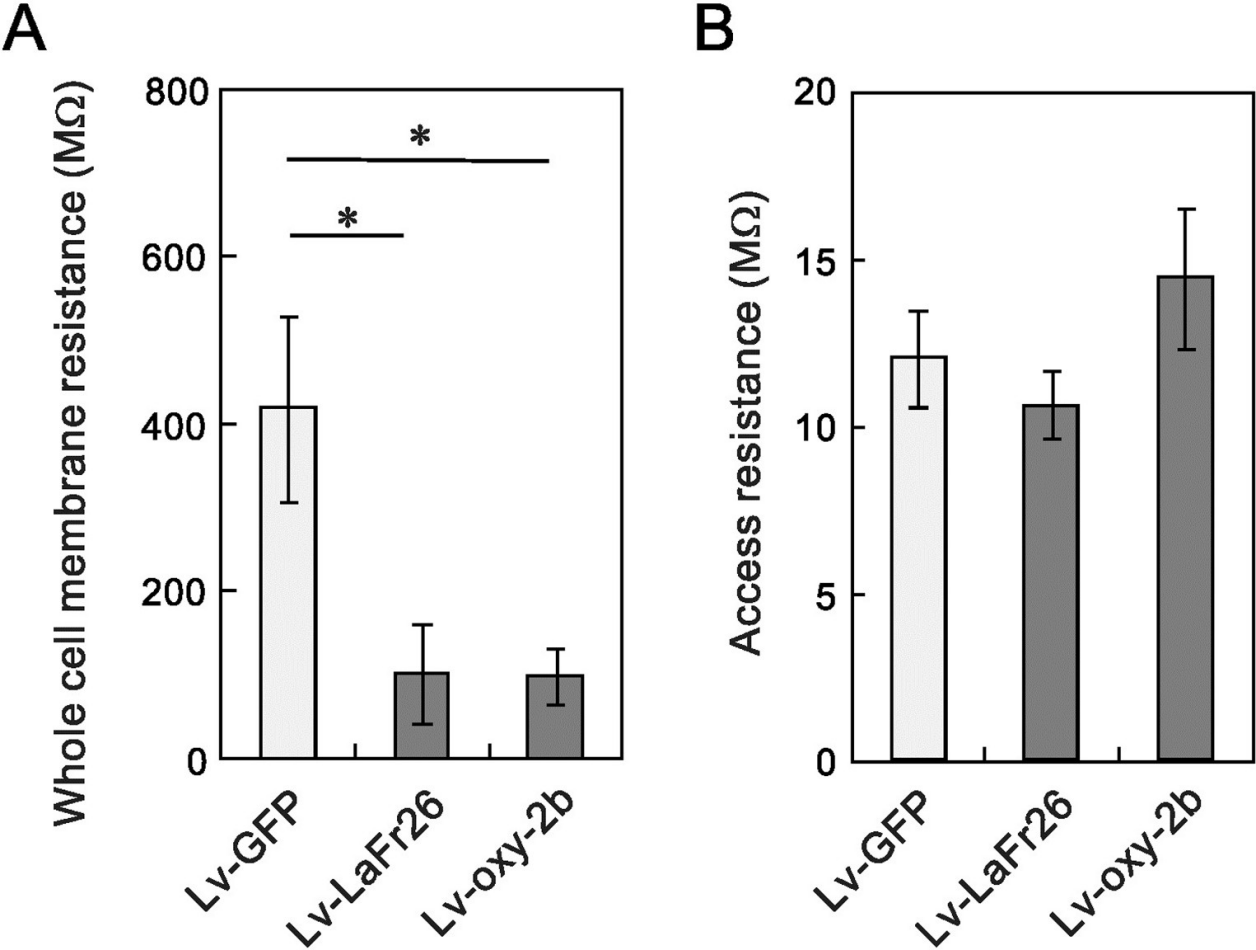
Decrease in the whole-cell membrane resistance in Lv-LaFr26 and Lv-Oxy-2b transduced cells. (A) Whole-cell membrane resistance was measured 48 h after transduction. Both Lv-LaFr26 and Lv-Oxy-2b decreased the resistance compared to that with Lv-GFP (n = 9, 9, and 6, *, p < 0.05 vs Lv-GFP treatment). (B) There was no difference in access resistance.

If secreted peptides were rapidly incorporated, pore should be formed. To confirm the pore formation in the cells transduced with Lv-LaFr26 and Lv-Oxy-2b, we measured whole-cell resistance with patch-clamp recordings from those cells and from the cells treated with the control Lv-GFP 48 h after transduction (Fig 8). If the pores were formed, the whole-cell resistance should be decreased. In fact, we have found a decrease in the whole-cell membrane resistance with chemically synthesized LaFr26 [11]. As expected, the whole-cell resistance of Lv-LaFr26 and Lv-Oxy-2b-treated cells was decreased, compared to those treated with Lv-GFP (Fig 8A). There was no difference in the access resistance of whole-cell recordings, indicating that the recording condition was the same (Fig 8B). At 72 h, the cell membranes were too leaky to obtain a stable patch-clamp recording from these transduced cells.

**Fig 9:**
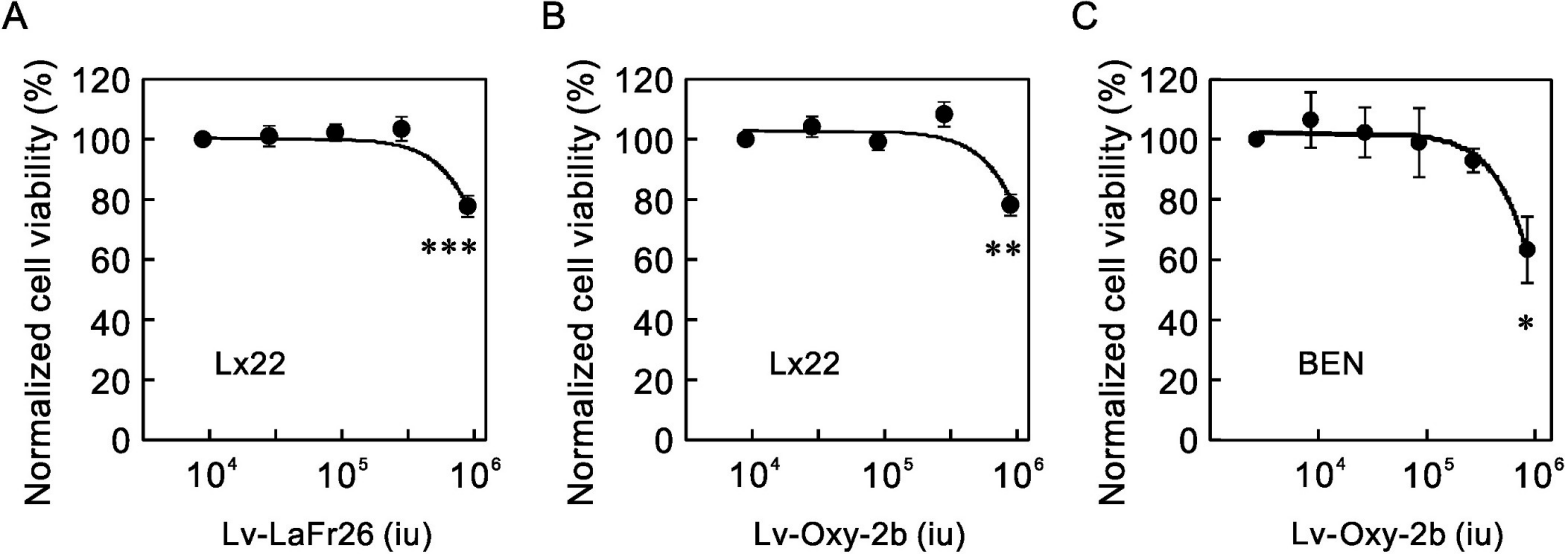
Cytotoxicity of Lv-LaFr26 and Lv-Oxy-2b to lung cancer cells. (A, B, and C) Lv-LaFr26 and Lv-Oxy-2b were added to the media of lung cancer cell lines, LX22 and BEN. The cell viabilities decreased (n = 4).

To confirm the cytotoxicity to cancer cells, which endogenously express K^+^ channels and are hyperpolarized, we added these lentiviral vectors to the media of LX22 and BEN cells and measured the viability (Fig 9). The transduction with Lv-LaFr26 and Lv-Oxy-2b decreased cell viability at the higher viral titers.

## Discussion

### K^+^ channel current- and hyperpolarization-dependent cytotoxicity of LaFr26 and oxyopinin-2b

We previously showed the selective incorporation of a spider venom peptide, LaFr26 (CIT1a), into Kir2.1 expressing cells, which resulted in neuronal depolarization and pain-related behavior in mice, suggesting that this peptide may be useful for the spider in deterring mammalian predators [11]. On the other hand, recent studies reported the upregulation of K^+^ channels expression in cancer cells [1, 2]. Here we showed the selective cytotoxicity of pore-forming spider venom peptides, LaFr26 and oxyopinin-2b, on K^+^ channel-expressing cells, including cancer cells. The K^+^ channel current dependency was confirmed by the lack of effect on the GFP-expressing 293T cells and the blockade of the toxicity by the inhibition of the K^+^ channel current. The correlation between cytotoxicity and the resting membrane potentials indicates its direct dependence on hyperpolarization. We expect that this approach could be applicable to cancer therapy. The key features that are required for safe and effective cancer therapy are tumor targeting and oncolytic activities. To favor these features, some ideas have been tried with venom peptides. For instance, a scorpion venom peptide, chlorotoxin, was shown to bind selectively to glioma cells through binding to matrix metalloproteinase-2 [21]. Although chlorotoxin has no oncolytic activity, it is now in clinical trials for detection of glioma cells during extirpation, using fluorescent-labeled chlorotoxin [22]. Alternatively, an idea that a latent pore-forming peptide, which would be selectively activated by metalloproteinases secreted by cancer cells, was also tried [23]. Similarly, we provide a new basis for targeted oncolytic activities which are dependent on the membrane potential of cells.

Nevertheless, the expression of K^+^ channel is upregulated in cancer cells [1, 2]. It is also reported that cancer cells tend to be more depolarized than their normal counterparts [24]. But this is not so in all cases. In fact, our results showed the lack of cytotoxicity to U87 and T98G cells, of which membrane potentials were depolarized, and the contrasting cytotoxicity to LX22 and BEN cells, which were hyperpolarized. Therefore, the effectiveness of these pore-forming peptides seems to be limited to hyperpolarized cancer cells. For instance, the peptides would be ineffective against cancer cells overexpressing HERG channels, which did not hyperpolarized membrane potential. Both LX22 and BEN cells are lung cancer cells of the neuroendocrine type, so it is possible that LaFr26 and oxyopinin-2b might be commonly effective on this cell type. In addition, as summarized in a review [24], prostate cancer cells are relatively hyperpolarized, and therefore these pore-forming peptides might be effective to prostate cancer too. On the other hand, the membrane potential was shown to be hyperpolarized at the G1-S transition [2, 24]. These spider venom peptides might be cytotoxic to the cells at the G1-S transition even in depolarized cancer cells. Thus, their effectiveness and side-effect seem to depend on the membrane potentials of the tumor and the surrounding normal cells. Reportedly, in glioblastoma cells, depolarizing Na^+^ and TRP channels are upregulated as well as K^+^ channels [25, 26]. It is also well known that the K^+^ channels are upregulated to compensation for the depolarization [27], and therefore K^+^ channels might be upregulated for compensation in glioblastoma cells. Further work will be needed to reveal the relationship between the membrane potential and the cytotoxicity of these pore-forming venom peptides.

### Cytotoxicity of lentiviral vectors expressing LaFr26 and oxyopinin-2b on K^+^ channel expressing cells

Despite their usefulness, cytotoxic venom peptides have drawback concerning their delivery as drugs for cancer therapy, as mentioned in the Introduction section. In order to overcome the drawback, here we provided a new basis for the antitumor therapy: the lentiviral expression of the pore-forming venom peptides in the tumor cells themselves. As expected, Lv-LaFr26 and Lv-Oxy-2b showed cytotoxicity to K^+^ channel expressing cells. The K^+^ channel current- and hyperpolarization-dependency was confirmed by the lack of cytotoxic effect on the GFP-expressing cells. The pore formation was confirmed by the decrease in the whole-cell membrane resistance.

We successfully detected the secretion of these peptides in the media using a cation exchange chromatography. A peak was detected at retention time which is close to that of the chemically synthesized peptide. But the retention time was a little bit longer than that of synthesized LaFr26: peak times were 19.8 and 18.9 min, respectively. It is likely that these peaks are LaFr26 for two reasons. First, LaFr26 is an extremely basic peptide: it consists of 20 basic and 6 acidic amino acids out of 69 amino acids, the isoelectric point is 10.6. It is unlikely that such extremely basic peptide was expressed in 293T cells. Second, there is no peak at around 19 min in the chromatogram of the medium from the control viral vector. Therefore, the elongation of the retention time might be attributable to a putative modification of the peptide produced by the viral vector-transduced cells. In the cation-exchange chromatography, the elongation in the retention time means that the modification made the peptide more basic, e.g., amidation of the C-terminus. It is also possible that the *Gaussia princeps* signal peptide was not correctly cleaved during translation and secretion process, and the extra amino acids made it basic. This possibility is unlikely because the sequence of the signal peptide fused to the N-terminus was neutral, MGVKVLFALICIAVAEA.

Interestingly, the peptide peak height was lower than that of chemically synthesized LaFr26 at 1 μM, which is a concentration lower than the cytotoxic level (EC_50_ 2.96 μM). In fact, the conditioned media had no cytotoxicity. This could indicate that the secreted peptides were probably incorporated into the surrounding cells’ membrane immediately after secretion, and thereby the concentration in the supernatant was lower than the cytotoxic level. Indeed, the whole-cell membrane resistance was decreased even before a decrease in cell viability was detected by the measurement with CCK-8. Thus, it is expected that intratumorally injection of Lv-LaFr26 and Lv-Oxy-2b will express these pore-forming peptides and kill the surrounding cancer cells without causing side effects to other hyperpolarized cells, such as cardiac and neural, and other types in remote places.

Gene therapy using lentiviral vectors is safe and is now approved by the American Food and Drug Administration for the treatment of acute lymphoblastic leukemia, and many lentiviral vectors are in clinical trials [28]. Moreover, several oncolytic virus therapies, which require intratumoral injection, are now trialed clinically [29]. The promoter can be replaced with a cancer cell-specific one, which should prevent the expression in normal cells and increase safety. If the expression of the peptides by the virus-transduced cells causes unwanted side-effects, this could be potentially canceled by engineering a suicide gene into the viral vector. In this way, the activation of the suicide gene will lead to the death of the transduced cells [30]. It can also be expected that the expression of venom peptides would induce an immune response since the peptides expressed by the transduced cancer cells can be recognized as nonself antigens. This immune response would probably enhance the oncolytic activity of the peptides, resulting in a synergistic effect. Thus, this study provides a new promising basis for cancer therapy. Further attempts will be applicable to increase in the potency, e.g., amino acid substitution of venom peptides, improvement of the construction of lentiviral vector, and tumor-selective pseudotyping of envelop protein.
